# Association between Polymorphisms in XRCC1 Gene and Treatment Outcomes of Patients with Advanced Gastric Cancer: A Systematic Review and Meta-Analysis

**DOI:** 10.1371/journal.pone.0085357

**Published:** 2014-01-21

**Authors:** Zhuo Cao, Jia Song, Jun Wang, Xufeng Guo, Shijie Yu, Weiguo Dong

**Affiliations:** Department of Gastroenterology, Renmin Hospital of Wuhan University, Wuhan, Hubei Province, PR China; Duke Cancer Institute, United States of America

## Abstract

**Background:**

Many reports have shown inconsistent results on the relationship between single nucleotide polymorphisms (SNPs) of X-ray repair cross complementing protein (XRCC1) gene and platinum-based chemotherapeutic efficacy. This meta-analysis aimed to summarize published data about the association between two SNPs of XRCC1 (Arg194Trp and Arg399Gln) and treatment outcomes of patients with advanced gastric cancer.

**Methodology/Principal Findings:**

We retrieved the relevant articles from MEDLINE, Web of Knowledge, and the China National Knowledge Infrastructure (CNKI) databases. Studies were selected according to specific inclusion and exclusion criteria. Study quality was assessed according to the guidelines outlined by Hayden, et al. and PRISMA guidelines. We estimated the odds ratio (OR) for response rate versus no response after platinum-based chemotherapy. Progression-free survival (PFS) and overall survival (OS) were evaluated by pooled Cox proportional hazard ratios (HRs) and 95% confidence intervals (CIs). We found that none of the XRCC1 Arg194Trp and Arg399Gln polymorphisms was significantly associated with tumor response. Stratified analysis by ethnicity or sensitivity analysis also showed that XRCC1 SNPs were not related with chemotherapy response. Patients with minor variant A allele were likely to have poorer 2-year survival rate than those with G/G genotype. However, in the group of 5-year follow up, there was no significant association between the A allele and OS yet.

**Conclusions/Significance:**

There is no evidence to support the use of XRCC1 Arg194Trp and Arg399Gln polymorphisms as prognostic predictors of TR and PFS in gastric patients treated with platinum-based chemotherapy. The relationship between minor variant A allele and OS requires further verification.

## Introduction

Worldwide, gastric cancer is the third most common cause of cancer death among males and the fifth in females [1]. Despite improvements in diagnosis and therapy, the overall survival time of advanced gastric cancer patients is still short. Platinum-based chemotherapy has been a common regimen for patients with advanced gastric cancer. However, chemotherapy sensitivity varied remarkably between different patients.

Up to now, researchers have determined that efficacy of the chemotherapy is multifactorial. Gene polymorphisms of drug target genes, genes involving in DNA repair pathways and detoxification pathways may influence the effect of the anti-cancer agents [2], [3].

XRCC1 gene repairs single-strand breaks by encoding a protein that defends breaks while repairs base excision through interacting with other proteins [4], [5]. Another study showed that XRCC1 protein could bind to platinum-containing DNA duplexes [6]. These studies imply that XRCC1 contributes to the repair of platinum-induced DNA damage. The single nucleotide polymorphisms in DNA repair pathways may alter gene expression and activity, therefore influence the effectiveness of cancer therapy and prognosis of patients [7]. The most extensively studied SNPs of XRCC1 gene are Arg399Gln (G > A, rs25487) and Arg194Trp (C > T, rs1799782). The two SNPs have been reported to be associated with an altered DNA repair activity [8], [9]. Therefore, these SNPs might alter the activity of DNA repair, thus influence the efficacy of platinum-based chemotherapy and the prognosis of patients.

Some researchers have studied the association between SNPs in XRCC1 gene and clinical outcome of gastric cancer patients [10]–[23]. However, the results were not consistent. We performed a systemic review and meta-analysis to assess the evidence about effects of XRCC1 SNPs on the efficacy of chemotherapy and overall survival in gastric cancer patients treated with platinum-based chemotherapy.

## Methods

### Retrieval of Published Studies

This meta-analysis focused on studies dealing with prognostic implication of XRCC1 SNPs in patients with gastric cancer. We searched for relevant publications before June 1^st^, 2013 by using electronic MEDLINE, Web of Knowledge and CNKI databases with the following terms “XRCC or X-ray repair cross complementing protein”, “gastric or stomach cancer”, “polymorphism or variant”, “chemotherapy or progression-free survival or overall survival”. We searched for studies without any language limitation. Furthermore, we screened the titles and abstracts to identify the relevant studies. The review was limited to the published studies and no contact was made with the authors to obtain unpublished data.

### Inclusion and Exclusion Criteria

The inclusion criteria were as follows: (1) patients with advanced, recurrent, or metastatic gastric cancer should be histologically or pathologically confirmed. (2) The gastric cancer patients were treated by any of the platinum drugs. (3) Studies should contain the information to estimate relative risks (i.e., ORs, HRs) and 95%CIs for prognostic effect of gastric cancer. (4) SNPs in XRCC1 gene should be genotyped. Unrelated articles and some types of original studies were not eligible for this meta-analysis, such as review, case report and meta-analysis. Studies were excluded if critical information was missing and not obtained by our repeated requests.

### Data Extraction

The following information was extracted from included publications: first author's name, year of publication, country, race of patients, source of patients, study design, number of patients, gender distribution, age (median), tumor stage, genotyping method, chemotherapy regimens, clinical outcomes, response criteria and genotype data.

### Quality assessment

The quality and risk of bias within the papers were critically appraised separately by two reviewers. Study quality was assessed according to the guidelines outlined by Hayden et al and PRISMA guidelines [24], [25]. For every included study, each of the following domains of potential bias was assessed:

1. Study participation: Inclusion and exclusion criteria defined in detail; The key characteristics of study population described in detail; Table sample size >50
2. Study attrition: Response rate >80%; Record of reason for loss to follow-up; No impact of loss to follow-up on the results of the study
3. Prognostic factor measurement: Genotyping methods fully described; Genotyping verified by sequence; Blindness of assessment for genotyping
4. Outcome measurement: WHO or RECIST criteria for tumor response; Outcome measure confirmed by repeat; Blindness of assessment for outcome
5. Confounding measurement and account: Adequately valid and reliable measurement used for all important confounders; Important potential confounders adjusted by multiple analysis
6. Analysis: Sufficient presentation of data to assess the adequacy of the analysis; No selective reporting of results.

Any differences in opinion were resolved by discussion, then by adjudication to a third reviewer. Studies of acceptable quality for inclusion in the synthesis would at least partly satisfy each of the six biases.

### Statistical methods

Hardy–Weinberg equilibrium (HWE) was calculated again using a goodness-of-fit test (χ^2^ or the Fisher exact tests, significant at the 0.05 level).

We estimated the OR for response rate versus no response after platinum-based chemotherapy [CR+PR vs. PD+SD, using the WHO criteria [26] or the Response Evaluation Criteria in Solid Tumors criteria (RECIST) [27].

The association between XRCC1 polymorphisms and reponse rate was estimated by calculating a pooled OR and 95% CI under four genetic models respectively (allele frequency: A vs. G; co-dominant model: A/A vs. G/G, G/A vs. G/G; dominant model: A/A+G/A vs. G/G; recessive model: A/A vs. G/G+G/A and complete overdominant model: A/A+G/G vs. G/A).

PFS and OS were evaluated by pooled Cox proportional HRs and 95% CIs using published methods [28]. HRs and 95% CIs were estimated directly from the raw [11], [13], [14], [20] or indirectly from the Kaplan–Meier curve of an article [17], [22].

We used the Cochran's Q test, with a significance level of P<0.05, to detect the between-study heterogeneity. We performed primary analyses with a fixed-effect model and confirmatory analyses with a random-effect model, if there was significant heterogeneity. We examined the effect of publication bias using inverted funnel plots and the Egger's test, and all analysis was carried using the Review Manager 5.2.

## Results

### Study Characteristics

**Figure 1:**
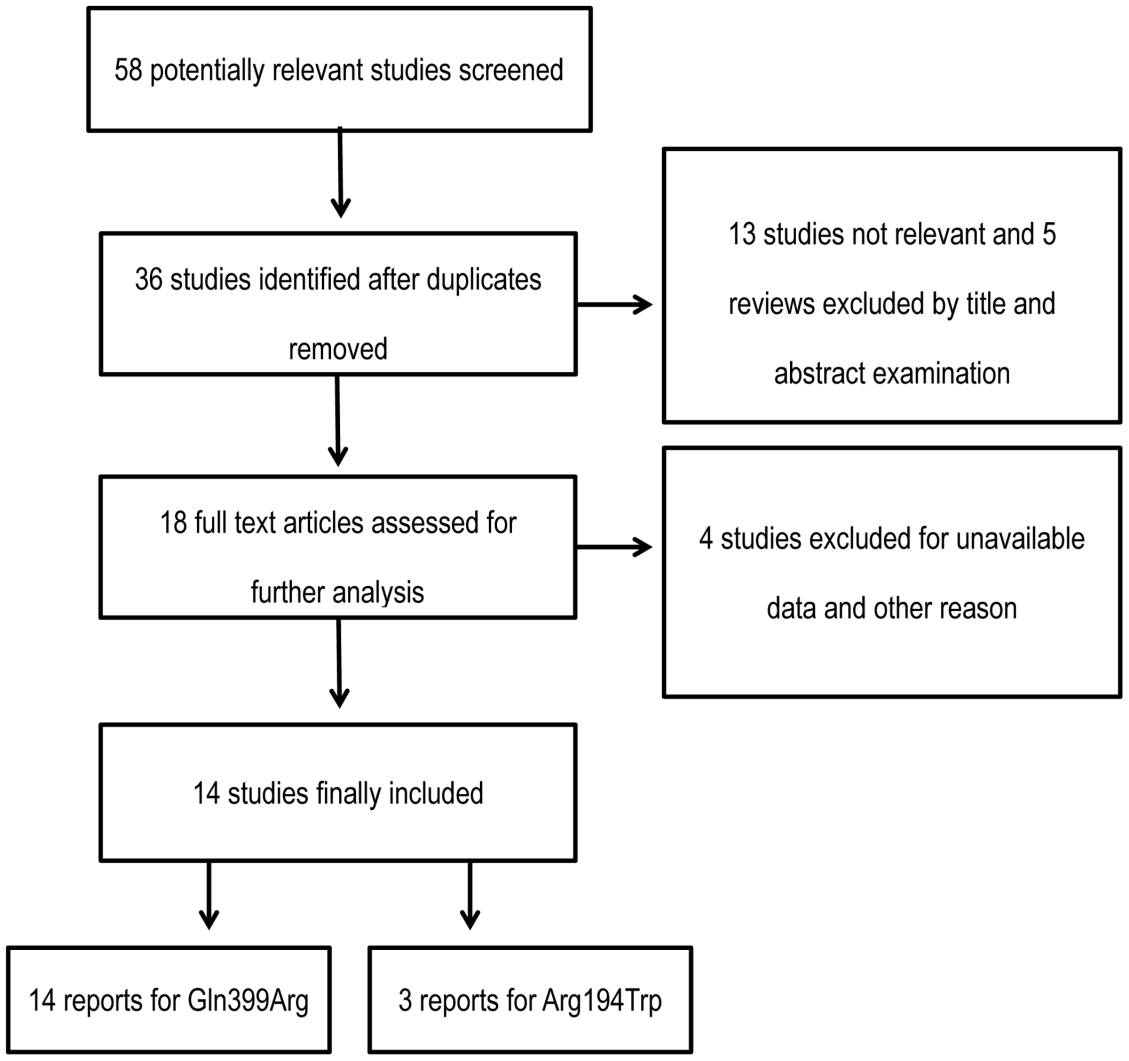
Flow diagram for study selection in meta-analysis.

Overall, 57 studies were selected during the first step of systematic literature review, of which 18 studies seemed to meet the inclusion criteria. Four studies were excluded because the data was inestimable and authors were unreachable [18], [29], [30], [31] (Fig. 1). Finally, the data pool consisted of 13 studies, including 1406 cancer patients.

Characteristics of the included studies are summarized in Table 1. Two of the included studies were conducted on Caucasian patients, and twelve were conducted on Asian patients. Ten studies were reported in English [10]–[13], [15]–[17], [19], [20], [22] and four were reported in Chinese [14], [21], [23]. The sample size ranged from 55 to 200 participants.

**Table 1:** Characteristics of included studies.

Stydy	Country	Source of patients	Race	NO. of patient (male%)	Age (mean)	Stage^a^	Chemotherapy (No.)^b^	Dose of platinum^c^	Study design^d^	Blindness of assessment^e^	Genotype data	Polymorphism detection method	Quality checks^f^	HWE	Outcomes^g^	Quality checks	mPFS (month)	mOS (month)
Liu Y 2011	China	Hospital	Asian	126(71.4%)	57	II-IV	mFOLFOX-4	L-OHP 85mg/m2 biweekly	U	U	Arg399Gln	TaqMan	Y	Y	OS	/	12	21
Park S R 2011	Korea	National Cancer Center	Asian	108(68.5%)	57	IV/R	S-1+DDP	DDP 60mg/m2 triweekly	P	U	Arg399Gln	PCR-RFLP	Y	N	TR/PFS/OS	Y	U	U
Ji M 2010	China	Hospital	Asian	59(67.8%)	35–75	IV	DCF	DDP 60mg/m2 triweekly	U	U	Arg399Gln	PCR-LDR	N	N	TR	N	/	/
Liang J 2010	China	Hospital	Asian	85(76.5%)	55	IV	L-OHP+CF+5-FU	L-OHP 130mg/m2 triweekly	P	U	Arg399Gln	TaqMan	N	Y	PFS*/OS*	/	5.3	8
Gao C 2010	China	Hospital	Asian	91(73.6%)	58	Advanced	CFL(30)/CFLH(10)/L-PF(32)/L-PFT(19)	L-OHP 100mg/m2 biweekly/100mg/m2 triweekly/DDP 6mg/m2	U	U	Arg399Gln/Arg194Trp	PCR-RFLP	N	Y	TR	N	/	/
Shim H J 2010	Korea	Hospital	Asian	200(75.0%)	58	IV/R	DDP+TAX(188)/ DDP+DOC(12)	DDP 75mg/m2 triweekly	P	U	Arg399Gln/Arg194Trp	PYRO/PCR-RFLP	N	Y	TR/PFS*/OS#	N	4.3	11.9
Won D Y 2010	Korea	Hospital	Asian	55(29.1%)	65	Advanced/R	mFOLFOX-6	L-OHP 100mg/m2 biweekly	P	U	Arg399Gln	PCR-RFLP	N	Y	TR/OS#	Y	5	14
Goekkurt E 2009	Germany	Multicenter	Caucasian	134(68.7%)	64	Metastatic	FLO(71)/FLP(63)	L-OHP 85mg/m2 biweekly/DDP 50mg/m2 biweekly	P	U	Arg399Gln	PCR-RFLP	Y	Y	TR*/PFS#/OS*	N	U	U
Qiu D 2009 Tahara T 2011	China Japan	Hospital Hospital	Asian Asian	68 130(68.5%)	55 65	IV Early/advanced	FOLFOX-4 TS-1/Taxane	L-OHP 130mg/m2 triweekly /	P P	U U	Arg399Gln Arg399Gln/Arg194Trp	PCR-LDR PCR-RFLP	N N	Y Y	TR/PFS OS	N /	8 /	/ 30
Huang Z 2009	China	Hospital	Asian	102(71.6%)	58	IB-IV	FOLFOX4(83)/ FOLFOX4+TAX/HCPT(19)	L-OHP 85mg/m2 biweekly /L-OHP 85mg/m2 biweekly	R	Y	Arg399Gln	PCR-LDR	N	Y	OS*	/	20	26
Keam B 2008	Korea	II clinical trial	Asian	73(65.8%)	59	IV/R	mFOLFOX-6	L-OHP 100mg/m2 biweekly	P	U	Arg399Gln	PCR-RFLP	N	Y	TR/PFS*/OS*	Y	6	12.6
Ruzzo A 2006	Italy	Multicenter	Caucasian	175(56.6%)	61	IV	FP	Unknown	P	Y	Arg399Gln/Arg194Trp	PCR-RFLP	N	Y	TR*/PFS#/OS#	U	6	9.8

Abbreviation:

a: R, recurrent.

b: DDP, cisplatin; S-1, Tegafur Gimeracil Oteracil Potassium Capsule; L-OHP, oxaliplatin; CF, calcium folinate; 5-FU, 5-fluoro-2,4 (1h, 3h) pyrimidinedione; TAX, paclitaxel; DOC, docetaxel; HCPT, hydroxycamptothecin.

c: U, unsure.

d: P, prospective; R, retrospective.

e: Y, yes.

f: N, no.

g: *, adjusted for confounders; #, data not given.

The quality assessment of the included studies is summarized in table 2. Of these studies, nine studies were prospective, one study was retrospective, and the rest did not specify this. All studies reported results on >80% of their patient sample. One study recruited patients from a national cancer center, nine recruited patients at hospital inpatient departments, two recruited cases from multiple medical centers, and one recruited patients as part of a II clinical trial.

**Table 2:** Quality assessment of included studies.

				Outcome measurement		Confounding measurement and account		Analysis	
Study	Study participants	Study attrition	Genotyping method	Response rate	PFS/OS	Response rate	PFS/OS	TR	PFS/OS
Liu Y 2011	Y	U	Y	/	U	/	Y	/	Y
Park SR 2011	Y	Y	Y	Y	Y	Y	Y	Y	Y
Ji M 2010	Y	Y	Y	P	/	N	/	Y	/
Liang J 2010	Y	U	Y	/	Y	/	Y	/	Y
Gao C 2010	P	Y	P	P	/	Y	/	Y	/
Shim HJ 2010	Y	Y	P	P	Y	Y	Y	Y	Y
Won DY 2010	P	U	P	Y	Y	Y	Y	Y	P
Goekkurt E 2009	Y	Y	Y	P	Y	Y	Y	Y	P
Huang Z 2009	Y	U	Y	/	Y	/	Y	/	Y
Tahara T 2011	Y	Y	P	/	Y	/	Y	/	Y
Qiu D 2009	P	N	P	P	P	N	N	Y	P
Keam B 2008	Y	U	Y	Y	Y	Y	Y	Y	Y
Ruzzo A 2006	Y	Y	P	P	Y	Y	Y	Y	P

Abbreviation: PFS: progression-free survival; OS: overall survival; Y: yes; P: partly; N: no; U: unsure.

Genotypes were verified by sequencing in all samples in two studies [17], [20], partially verified in 20% of samples in one study [12], and not verified in the rest of the studies.

Tumor responses were evaluated using WHO criteria or RECIST criteria. Response rate was confirmed in three studies [11], [16], [17] and not confirmed in the rest of the studies. Clinical investigators were blind to the results of genotyping in only two studies [10], [13] and in the remaining eleven studies this information was not reported.

Potential confounders were fully reported in nine out of 13 identified studies. Statistical analysis was adjusted for confounding variables in seven studies for clinical outcomes.

The frequency for 194Trp was from 6.9% to 32.5% with respect to response rate and that for 399Gln was from 18.0% to 42.4% in Chinese patients and 36.0% to 38.0% in Caucasian patients (Table 3).

**Table 3:** Allele frequency of XRCC1 Arg194Trp and Arg399Gln.

	Arg399Gln				Arg194Trp			
Study	ArgArg	ArgGln	GlnGln	allele frequency %(Gln)	ArgArg	ArgTrp	TrpTrp	allele frequency %(Trp)
Liu Y 2011	71	33	6	20.5	-	-	-	-
Park SR 2011	49	38	21	37.0	-	-	-	-
Ji M 2010	25	18	16	42.4	-	-	-	-
Liang J 2010	46	28	7	25.9	-	-	-	-
Gao C 2010	46	35	10	30.2	42	41	8	31.3
Shim HJ 2010	101	88	11	27.5	153	20	2	32.5
Won DY 2010	37	16	2	18.0	-	-	-	-
Goekkurt E 2009	52	61	20	38.0	-	-	-	-
Huang Z 2009	38	24	6	26.5	-	-	-	-
Tahara T 2011	65	51	12	29.3	-	-	-	-
Qiu D 2009	62	35	5	22.1	-	-	-	-
Keam B 2008	48	21	4	20.0	-	-	-	-
Ruzzo A 2006	71	82	22	36.0	85	100	15	6.9

Using the frequencies of XRCC1 genotypes, all populations were found to be in HWE except two studies by Ji M et al and Park SR et al [17], [19]. Minelli C et al. recently pointed out that studies that appear to deviate from HWE should be investigated further rather than just excluded unless there are other grounds for doubting the quality of the study [32]. In our meta analysis, the HWE-deviant population evaluated by Park SR was not excluded because no genotyping error was detected by PCR-RFLP combined with sequencing [17], while that population evaluated by Ji M was excluded from the study because of small samples and low quality [19].

### Response rate of XRCC1 Arg194Trp polymorphism

Because the study on the association between XRCC1Arg194Trp polymorphism with PFS or OS was too few to be analyzed, we only analyzed the association of XRCC1 Arg194Trp polymorphism with tumor response in gastric cancer patients in this meta-analysis.

Three studies with a total sample size of 466 patients were eligible for the final analysis. The results from the meta-analysis indicated no statistically significant association between XRCC1 Arg194Trp polymorphism and tumor response under all the genetic models (T vs. C: OR = 1.15, 95% CI 0.83–1.61; dominant model: OR = 1.23, 95% CI 0.80–1.87; recessive model: OR = 1.11, 95% CI 0.48–2.58)(Table 4). No significant publication bias was detected by either the inverted funnel plot or Begg's test (data not shown).

**Table 4:** Analysis of the association between XRCC1 Arg194Trp and response rate in different models.

Genetic models	Fixed-effect model (95%CI)	P1	I^2^	P2
T vs. C	1.15(0.83, 1.61)	0.41	0%	0.73
C/T vs. C/C	1.23(0.79, 1.91)	0.36	0%	0.93
T/T vs. C/C	1.22(0.51, 2.93)	0.66	10%	0.33
T/T+C/T vs. C/C	1.23(0.80, 1.87)	0.34	0%	0.99
T/T vs. C/T+C/C	1.11(0.48, 2.58)	0.81	23%	0.27
T/T+C/C vs. C/T	0.84(0.55, 1.28)	0.41	0%	0.71

Abbreviation: P1, p value for difference; P2, p value for heterogeneity.

### Response rate of XRCC1 Arg399Gln Polymorphism

Eight studies including 903 patients were qualified for the final analysis.

**Figure 2:**
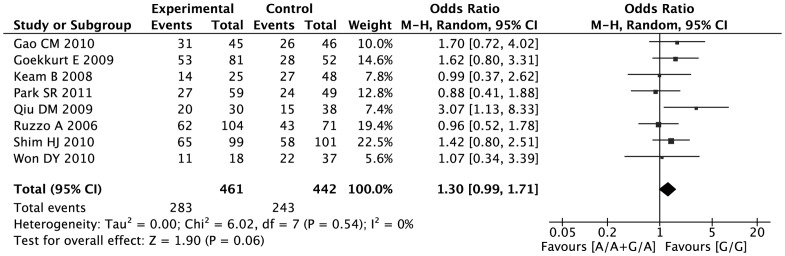
Forest plots of response rate in AGC patients treated with chemotherapy by XRCC1 Arg399Gln polymorphism: G/A or A/A vs. G/G.

The results from the meta-analysis indicated no statistically significant association between XRCC1 Arg399Gln polymorphism and tumor response under all the genetic models (figure 2) (A vs. G: OR = 1.17, 95% CI 0.94–1.46; dominant model: OR = 1.30, 95% CI 0.99–1.71; recessive model: OR = 1.19, 95% CI 0.74–1.90; (Table 5), and no single study altered the result substantially by the sensitivity test. Stratified analysis by ethnicity showed the 399Gln allele was not associated with response rate rate neither in Asians or in Caucasians. No significant publication bias was detected by either the inverted funnel plot or Begg's test (data not shown).

**Table 5:** Stratified analysis of the association between XRCC1 Arg399Gln and response rate.

Study groups	No. studies	OR, 95%CI)	*P1*	*I^2^*	*P2*	No. studies	OR, 95%CI)	*P1*	*I^2^*	*P2*	No. studies	OR, 95%CI)	*P1*	*I^2^*	*P2*
	**A vs. G**					**A/A+G/A vs. G/G**					**A/A vs. G/G+G/A**				
All	6	1.17[0.94,1.46]	0.16	0%	0.97	8	1.30[0.99,1.71]	0.06	0%	0.54	6	1.19[0.74,1.90]	0.47	0%	0.63
Caucasians	2	1.12[0.80,1.57]	0.51	0%	0.94	2	1.21[0.73,2.01]	0.46	16%	0.28	2	1.06[0.33,3.35]	0.93	64%	0.09
Asians	4	1.21[0.90,1.62]	0.20	0%	0.84	6	1.36[0.97,1.89]	0.07	0%	0.46	4	1.33[0.70,2.54]	0.38	0%	0.95
	**G/G+A/A vs. G/A**					**G/A vs. G/G**					**A/A vs. G/G**				
All	6	0.88[0.61,1.28]	0.51	33%	0.19	6	1.29[0.86,1.65]	0.28	8%	0.36	6	1.30[0.79,2.14]	0.30	0%	0.92
Caucasians	2	0.81[0.29,2.31]	0.70	80%	0. 03	2	1.27[0.53,3.07]	0.59	67%	0.08	2	1.38[0.70,2.71]	0.35	0%	0.93
Asians	4	0.89[0.60,1.30]	0.54	0%	0.47	4	1.19[0.80,1.77]	0.39	0%	0.50	4	1.21[0.58,2.53]	0.35	0%	0.61

Abbreviation: *P1*, p value for difference; *P2*, p value for heterogeneity.

### Progression free survival and Overall survival of XRCC1 Arg399Gln Polymorphism

**Figure 3:**
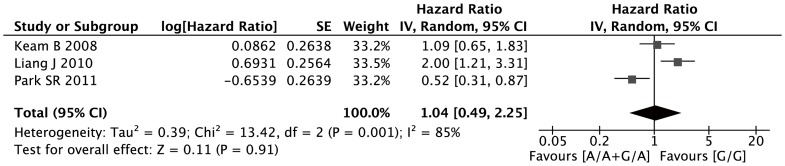
Forest plots of PFS in AGC patients treated with chemotherapy by XRCC1 Arg399Gln polymorphism: G/A or A/A vs. G/G.

Three studies were eligible for analyzing the relationship between the minor variant A allele and progression-free survival. In dominant model, the XRCC1 399 A allele was not associated with high risks of disease progression for gastric cancer patients (G/A + A/A versus G/G: HR, 1.04; 95%CI, 0.49–2.25; I^2^ = 85%, p = 0.001 for heterogeneity) (Fig. 3).

**Figure 4:**
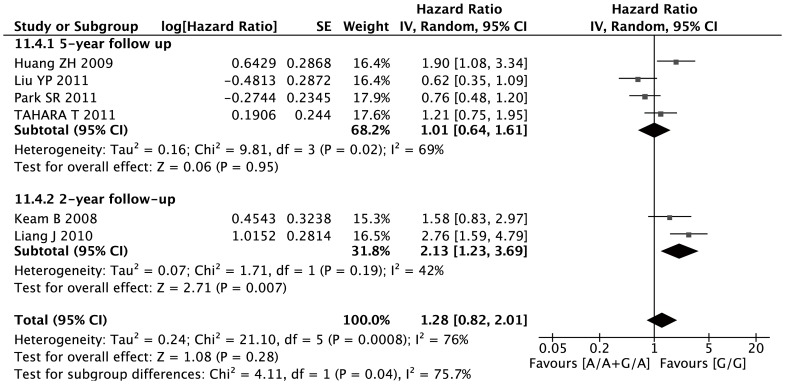
Forest plots of OS in AGC patients treated with chemotherapy by XRCC1 Arg399Gln polymorphism: G/A or A/A vs. G/G, stratified by follow-up time.

Six studies with a total number of 569 patients were eligible for the final analysis. In dominant model, the XRCC1 Arg399Gln SNPs was not associated with increasing risk of death in all patients (G/A+A/A versus G/G: HR, 1.28; 95%CI, 0.82–2.01; I^2^ = 76%, p = 0.007 for heterogeneity) (Fig. 4).

There was significant heterogeneity when these six studies were combined. Stratified analysis by follow-up time, the A allele was associated with more risk of death in the subgroup of 2-year follow up(G/A or A/A versus G/G: HR, 2.32; 95%CI, 1.72–3.13; I^2^ = 0%, p = 0.38 for heterogeneity) (Fig. 4). However, there was no significant association between the A allele and survival in the group of 5-year follow up. No significant publication bias was detected by either the inverted funnel plot or Begg's test (data not shown).

## Discussion

In this meta-analysis, there was not any evidence for an association nor an ethnic difference between the XRCC1 194 and 399 polymorphisms and tumor response in all patients. However, the minor variant A allele of XRCC1 399 polymorphism was negatively associated with progression-free survival and 2-year survival in gastric cancer patients.

Platinum agents are activated intracellularly, covalently binding to DNA to induce DNA adducts and finally leading to cell death. Various signal transduction pathways, including DNA damage recognition and repair, cell-cycle arrest and cell apoptosis, involves in this process to exert anticancer effects. Cancer cells with enhanced ability to repair DNA damage caused by platinum agents, may be resistant to the chemotherapy. There is evidence that cancer patients with a lower DNA repair capacity had an increased overall survival after platinum-based chemotherapy [33], [34].

X-Ray repair cross-complementing groups are important proteins of the DNA repair pathways. The XRCC1 protein associates with other proteins to facilitate the processes of base excision repair or single-strand break repair [5]. XRCC1 SNPs have been reported to be associated with an altered DNA repair activity [33], [34]. Arg194Trp and Arg399Gln are the most common SNPs in XRCC1 gene, and the 399Gln polymorphism was considered of increasing chemotherapy sensitivity [35].

With a pooled dataset of 903 patients treated with platinum-based regimens, we made a comprehensive assessment of prognosis of gastric cancer patients by response rate, PFS and OS. We found 399Gln allele of XRCC1 polymorphism was negatively associated with the response rate in all patients treated by platinum-based chemotherapy. We found neither Arg194Trp nor Arg399Gln of XRCC1 polymorphism influenced the response rate in all patients treated by platinum-based chemotherapy. No single study altered the result substantially by the sensitivity test. In further stratified analysis by ethnicity, significant association between XRCC1 399Gln allele and response rate was not detected in any of the subgroups yet. The common negative result of subanalysis and sensitivity test provided evidence that there may be no correlation regarding to XRCC1 399 Gln polymorphism and Platinum based chemotherapy result in the cases of gastric cancer patients.

This result is inconsistent with the conclusion of previous meta-analysis on predictive value of XRCC1 SNPs in patients with various cancers, such as lung cancer, colorectal cancer, and so on [36]–[38]. Polychemotherapy is the established form of treatment in advanced gastric cancer. The effect of chemotherapy is carried forward through a multigenic cascade. Therefore, other genetic variations that influence the tolerance to DNA adducts, function of DNA repair and drug metabolism may be more significantly associated with response rate than XRCC1 gene.

Initially, we found that the minor variant A allele was not obviously associated with progression-free survival and overall survival. Heterogeneity was detected in the analysis of XRCC1 Arg399Gln to overall survival, which indicated variability. Heterogeneity may have been caused by different characteristics, such as ethnicity, tumor stage, sample size, or follow-up time [39]. By subanalysis on follow-up time, there was significant subgroup difference between studies followed up by 2-year and 5-year (P = 0.04). The minor variant A allele was obviously associated with poor OS in studies with 2-year follow up, while not in the studies with 5-year follow up. However, heterogeneity was not removed yet by subanalysis. Our findings suggested that the effect of XRCC1 Arg399Gln polymorphism in clinical outcomes might need to be explored more carefully in future studies incorporating more criteria in the design and experimentation to ensure a more accurate and robust conclusion.

There were several limitations in our meta-analysis. Firstly, the total sample size for analysis of association between progression-free survival and overall survival and XRCC1 SNPs was small, therefore, subgroup analysis for influence of Arg194Trp on response rate and Arg399Gln on PFS and OS could not be performed in the present meta-analysis. Also, some of the findings in subgroups may have been undervalued because of the smaller sample size available for analyses. Secondly, significant heterogeneity between-study was obtained. Most of the eligible studies differed significantly in the study designs, such as patient selection, chemotherapeutic protocol and follow-up time. These may have caused significant heterogeneity between studies. Thirdly, our analysis used published international studies, of which studies [18], [29]–[31] was excluded from the analysis because of loss of contact for original data. We did not include the data of overall survival of XRCC1 Arg399Gln from the study by Shim HJ et al, Goekkurt E et al and Ruzzo A et al [10], [12], [15], because we can't get required information to estimate HR for 399Gln allele neither from the raw or indirectly from the Kaplan–Meier curve of an article. This could cause some bias in our estimates, but it is unlikely to change our main conclusions. In addition, we were unable to analyze the association between XRCC1 SNPs and platinum adverse effects, because few studies provided this information.

## Conclusions

Genetic polymorphisms in XRCC1 gene were not likely to be associated with response to platinum-based chemotherapy in advanced cancer patients. However, the relationship between XRCC1 SNPs and overall survival need larger sample size studies to make a further confirmation.

## Supporting Information

Checklist S1 **PRISMA checklist.**
(DOC)
